# Refining S-acylation: Structure, regulation, dynamics, and therapeutic implications

**DOI:** 10.1083/jcb.202307103

**Published:** 2023-09-27

**Authors:** Muhammad U. Anwar, F. Gisou van der Goot

**Affiliations:** 1 Global Health Institute, School of Life Sciences, École Polytechnique Fédérale de Lausanne, Lausanne, Switzerland

## Abstract

With a limited number of genes, cells achieve remarkable diversity. This is to a large extent achieved by chemical posttranslational modifications of proteins. Amongst these are the lipid modifications that have the unique ability to confer hydrophobicity. The last decade has revealed that lipid modifications of proteins are extremely frequent and affect a great variety of cellular pathways and physiological processes. This is particularly true for S-acylation, the only reversible lipid modification. The enzymes involved in S-acylation and deacylation are only starting to be understood, and the list of proteins that undergo this modification is ever-increasing. We will describe the state of knowledge on the enzymes that regulate S-acylation, from their structure to their regulation, how S-acylation influences target proteins, and finally will offer a perspective on how alterations in the balance between S-acylation and deacylation may contribute to disease.

## Teaser

S-acylation is one of the most frequent posttranslational modifications that regulates diverse cellular processes. Anwar and van der Goot discuss the complexity and disease implications of this highly regulated reversible lipidation.

## Introduction

Posttranslational modifications (PTMs) involve the reversible or non-reversible structural modifications of a protein (Lee et al., 2023). Proteome-wide data analysis suggests that there are about 400 different PTMs that can affect different aspects of protein structure, localization, and function (Khoury et al., 2011; Ramazi and Zahiri, 2021). By the enzymatic addition of functional groups, these modifications often alter the physico-chemical properties, such as local charge (Duan and Walther, 2015). Within the scope of this review, we will focus on lipid modifications, which alter protein hydrophobicity (Resh, 2013). These include N-terminal myristoylation (Khandwala and Kasper, 1971), prenylation (Wang and Casey, 2016), i.e., farnesylation and geranylgeranylation, and different forms of acylation (Zaballa and van der Goot, 2018).

While we will briefly mention these various lipid modifications, the major topic of this review is S-acylation, due to its reversibility (Jiang et al., 2018; Zaballa and van der Goot, 2018) and its extremely high occurrence in eukaryotes (Khoury et al., 2011), affecting 10–20% of the human proteome (https://Swisspalm.org; Blanc et al., 2015). Recent research has shown that S-acylation affects most major cellular signaling pathways such as Wnt (Abrami et al., 2008), Hippo (Noland et al., 2016), mTOR (Sanders et al., 2019), Ras/MAPK (Swarthout et al., 2005), Akt (Blaustein et al., 2021), and epidermal growth factor receptor (EGFR; Bollu et al., 2015). The ability to remove the lipid offers the option of acting as an on/off-switch to fine-tune the properties of target proteins, as is the case of phosphorylation (Nishi et al., 2014) or ubiquitination (Pickart and Eddins, 2004). We will discuss the various enzymes involved in S-acylation and deacylation, the dynamic nature of the modification, and how the cells achieve a multilayered regulatory control on these enzymes to ensure physiological cellular S-acylation. Finally, we will describe how impaired S-acylation is emerging as a significant contributor to a variety of diseases, ranging from neurological disorders to defective immune function and cancer. These findings, combined with the elucidation of the structures of acylation–deacylation enzymes, are opening new therapeutic perspectives.

### Lipid posttranslational modifications

Lipid PTMs involve the addition of lipophilic groups on proteins, thereby increasing their hydrophobicity and affinity toward cellular membranes or membrane domains (Jiang et al., 2018; Levental et al., 2010). At least five different lipophilic groups can be added, each conferring distinct properties to a target protein (Jiang et al., 2018). While all lipid modifications affect the lipophilicity of target proteins, the differences in lipid structure influence their membrane affinity and subcellular and even submembrane localization (Levental et al., 2010). Not only do the different types of lipid modifications amplify the cellular proteome but also some proteins combine multiple lipid modifications, such as myristoylation and S-acylation, to tune their membrane-binding properties in time and space (Alland et al., 1994; Galbiati et al., 1999; Zha et al., 2000).

#### Myristoylation

Myristoylation is the irreversible attachment of a 14-carbon saturated acyl group to the N-terminal glycine residue of a protein (Jiang et al., 2018; Zha et al., 2000). Since proteins are not synthesized with an N-terminal glycine, this modification requires a prior proteolytic step. Based on the mechanism of generation of N-terminal glycine, N-myristoylation can occur either co-translationally (when an aminopeptidase removes the starting methionine of a substrate to liberate an N-terminal Gly-Xxx-Ser/Thr/Cys motif at the ribosome) or posttranslationally (when an N-terminal glycine is exposed through proteolytic cleavage at an internal site of the protein; Boutin, 1997; Johnson et al., 1994; Martin et al., 2011). Subsequently, the N-myristoyltransferase (NMT) forms an amide bond to anchor the myristoyl to the protein. Myristolyation may also occur following proteolytic processing of substrates by caspases during apoptosis (Magee and Seabra, 2005; Resh, 1999). Myristoylation affects subcellular localization, protein–protein interactions, and protein function, thus playing important roles in signal transduction, cell death, and antimicrobial response (Yuan et al., 2020; Wang et al., 2021). Due to its broad involvement in cellular processes, molecular inhibition of NMTs has been explored for therapeutic applications, particularly in the context of cancer (Beauchamp et al., 2020).

#### Prenylation

Prenylation is the covalent attachment of a preassembled lipid consisting of either several farnesyl (branched 15-carbon) or several geranylgeranyl (branched 20-carbon) isoprene units to a free thiol of a cysteine side chain at, or near, the C-terminus of a protein via formation of a thioether bond by the action of protein prenyltransferases (Jiang et al., 2018; Wang and Casey, 2016). This modification is frequent in the Ras superfamily of small GTPases (Farnsworth et al., 1994), which play major roles in membrane trafficking, cell signaling, cancer (Hancock et al., 1989), and other pathologies including Alzheimer’s disease (Jeong et al., 2018).

#### Acylation

Medium/long acyl-chains such as palmitate (C16) can be covalently attached to different amino acids: N-acylation on lysines (James et al., 2020), O-acylation on serines (Yang et al., 2008), and S-acylation on cysteines (Jiang et al., 2018; Zaballa and van der Goot, 2018). In addition to different types of chemistry, these modifications can occur in topologically different environments, i.e., O-acylation of Wnt and Hedgehog proteins by membrane-bound O-acyltransferase (MBOAT) occurs in the lumen of the ER (Kohtz et al., 2001; Rios-Esteves et al., 2014) while S-acylation occurs on the cytosolic side of membranes (Linder and Deschenes, 2007; Fukata and Fukata, 2010; Zaballa and van der Goot, 2018; Salaun et al., 2020).

**Figure 1.**
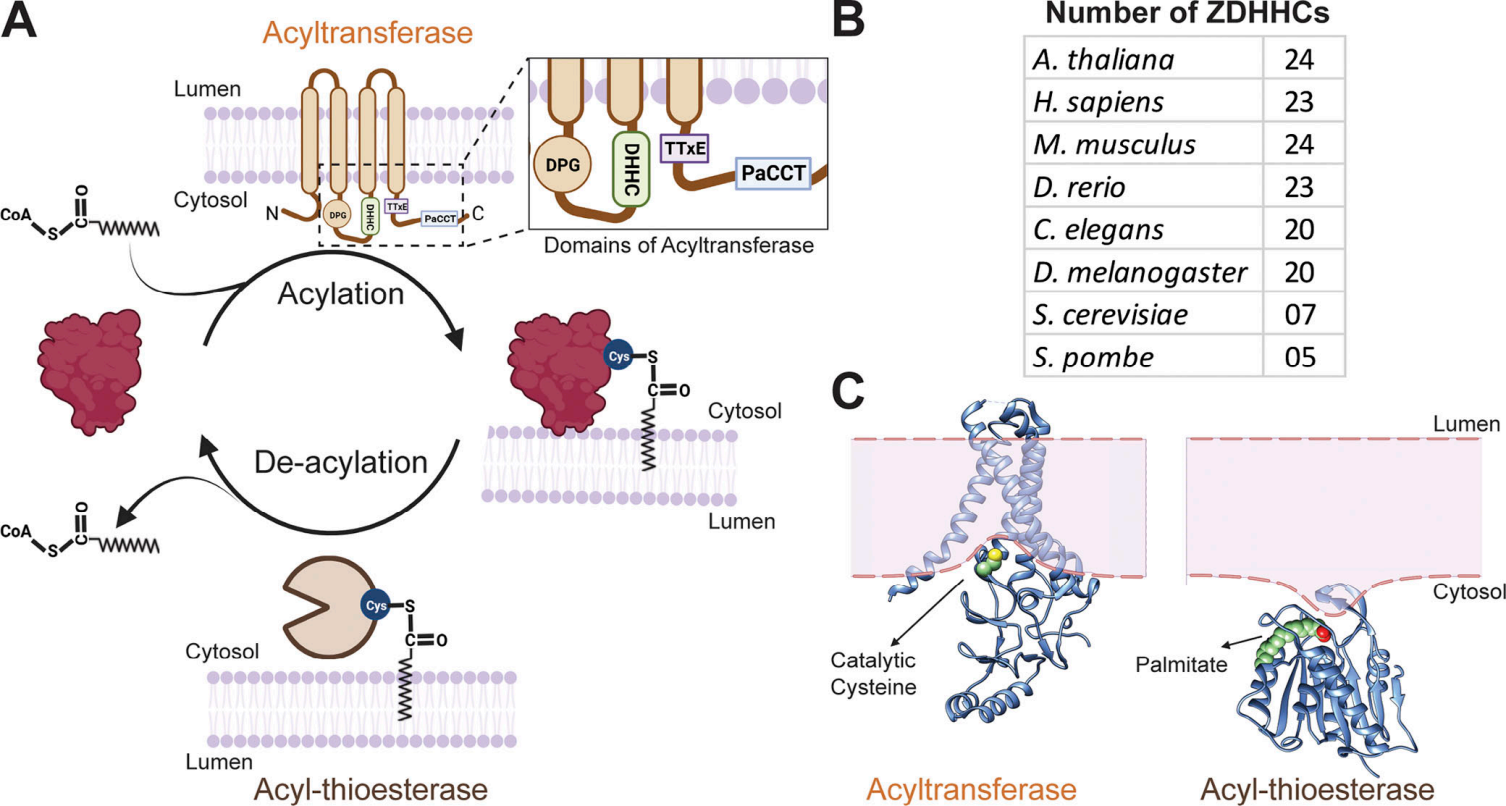
**The protein S-acylation cycle. (A)** Schematic representation of the S-acylation cycle. A medium-long chain fatty acid moiety from an acyl-CoA is added to a cytosolic cysteine of the target protein by an acyltransferase (ZDHHC). The enlarged view shows different domains of an acyltransferase. The thioester linkage formed between the acyl chain and the thiol group is hydrolyzed by an acyl-protein thioesterase (APT). **(B)** Number of ZDHHC acyltranferases in different model organisms. **(C)** Structures of acylating and deacylating enzymes. Left panel: Ribbon diagram of ZDHHC20, schematically showing that it leads to membrane deformation to expose its catalytic site to the cytosolic milieu (Stix et al., 2020b). Right panel: Ribbon diagram of APT2, schematically showing that it deforms the lipid monolayer to which it binds, facilitating the extraction of the acyl chain, which is covalently attached to the APT2 substrate, from the membrane for hydrolysis (Abrami et al., 2021). The extracted acyl chain is shown in the green space field.

S-acylation is the most frequent lipid modification. Since palmitate (C16) is most often the attached acyl chain, S-acylation is also referred to as S-palmitoylation (Schmidt and Schlesinger, 1979). It is mediated by a family of transmembrane protein acyltransferases, the ZDHHCs, and the fatty acid can subsequently be removed by acyl-proteins thioesterases (APTs; Fig. 1 A; Bheda et al., 2016; Jing et al., 2017).

### The S-acylating and de-acylating enzymes

S-acylation operates in cycles of acylation and de-acylation (Fig. 1 A), the speed of which can differ vastly amongst proteins, from minutes to many hours (Linder and Deschenes, 2007; Zaballa and van der Goot, 2018). Both addition and removal of acyl chains can be regulated as discussed later.

#### ZDHHC enzymes

**Figure 2.**
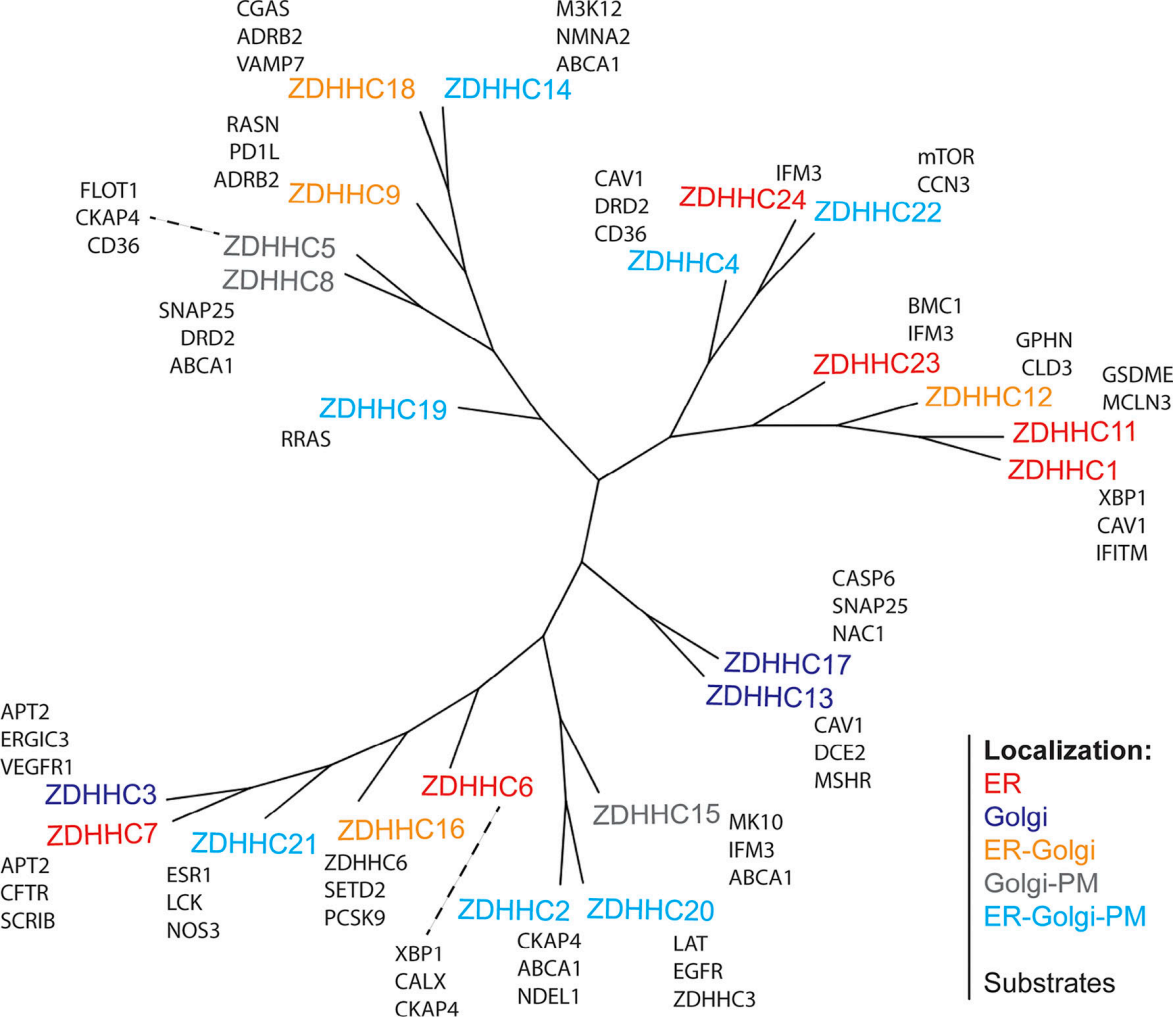
**Phylogenetic tree of human ZDHHC enzymes.** Multiple sequence alignment of human ZDHHCs was performed using Clustal Omega (Sievers et al., 2011). The tree was constructed using the neighbor-joining method and was visualized using iTOL (Letunic and Bork, 2011). ZDHHCs are color-coded based on their reported subcellular localization(s) (Abrami et al., 2017, 2021; Ernst et al., 2018; Sandoz et al., 2023), and examples of substrates for each enzyme are shown in black (for a complete list of reported S-acylated proteins visit https://www.swisspalm.org/ [Blanc et al., 2015]).

ZDHHC enzymes are found in all eukaryotes (Fig. 1 B). Intriguingly, the number of different ZDHHCs remains rather similar, ranging between 15 and 24 as organismal complexity increases from flies to humans (Fig. 1 B and Fig. 2; Bannan et al., 2008; Edmonds and Morgan, 2014). ZDHHCs are ubiquitously expressed, even though expression levels may vary between tissues. They are, however, low-abundance enzymes as indicated by quantitative proteomics studies (Table 1 and Table S1; Beck et al., 2011; Bekker-Jensen et al., 2017; Hein et al., 2015; Nagaraj et al., 2011). In HeLa cells, for example (Table 1), 17 out of the 23 ZDHHC enzymes are present at <3,000 copies per cell (as a reference point, a cell contains ∼32 million copies of GAPDH). The two enzymes that stand out are ZDHHC3 (11,000 copies per cell) and ZDHHC5 (60,000 copies per cell). Given that 10–20% of the human proteome may undergo acylation (https://SwissPalm.org), with a total of <150,000 copies of ZDHHC enzymes in cells, these enzymes must be highly efficient and stringently regulated.

**Table 1.** Abundance of S-acylation and deacylation enzymes

Acyltransferases (ZDHHCs), HeLa cells		
Enzyme	Uniprot AC	Copies/Cell
zDHHC1	Q8WTX9	
zDHHC2	Q9UIJ5	1,631
zDHHC3	Q9NYG2	11,327
zDHHC4	Q9NPG8	
zDHHC5	Q9C0B5	60,617
zDHHC6	Q9H6R6	4,568
zDHHC7	Q9NXF8	2,737
zDHHC8	Q9ULC8	2,321
zDHHC9	Q9Y397	1,598
zDHHC11	Q9H8X9	
zDHHC12	Q96GR4	928
zDHHC13	Q8IUH4	4,751
zDHHC14	Q8IZN3	1,552
zDHHC15	Q96MV8	
zDHHC16	Q969W1	
zDHHC17	Q8IUH5	6,380
zDHHC18	Q9NUE0	1,712
zDHHC19	Q8WVZ1	
zDHHC20	Q5W0Z9	7,986
zDHHC21	Q8IVQ6	2,879
zDHHC22	Q8N966	
zDHHC23	Q8IYP9	630
zDHHC24	Q6UX98	
**Acyl-protein thioesterases (APTs), HeLa cells**		
LYPLA1 (APT1)	O75608	527,114
LYPLA2 (APT2)	O95372	715,617
PPT1	P50897	1,035,263
PPT2	Q9UMR5	70,881
ABHD2	P08910	4,273
ABHD3	Q8WU67	1,825
ABHD4	Q8TB40	9,544
ABHD5	Q8WTS1	9,921
ABHD6	Q9BV23	21,560
ABHD10	Q9NUJ1	197,249
ABHD11	Q8NFV4	157,176
ABHD12	Q8N2K0	55,135
ABHD13	Q7L211	4,159
ABHD14A	Q9Y3T7	2,545
ABHD14B	Q96IU4	607,032
ABHD15	Q96EC5	3,469
ABHD16A	O95870	25,149
ABHD17A	Q96GS6	27,342
ABHD17B	Q5VST6	21,215
ABHD17C	Q6PCB6	2,735

The copy number of proteins in cells can be determined by quantitative proteomics approaches. Several studies have reported the abundance of ZDHHC enzymes and acyl protein thioesterases (see Table S1). Here, we show the abundance reported by the latest of these studies (Bekker-Jensen et al., 2017), which, probably due to the improvement of the mass spectrometry sensitivity, provides quantification for the highest number of these enzymes. In a number of studies, ZDHHC enzymes are measured but their abundance was considered below what was quantifiable, i.e., <700 to 1,000 copies per cell.

ZDHHC enzymes are membrane proteins, with at least four transmembrane domains (TMDs), as recently reviewed (Gottlieb and Linder, 2017; Stix et al., 2020a). ZDHHC4 and 24 may however contain five TMDs, while ZDHHC13, 17, and 23 are predicted to have six (Zaballa and van der Goot, 2018). The catalytic motif is located within the cytosolic cysteine-rich domain (CRD) between TMD2 and TMD3 (Fig. 1, A and C). Other conserved regulatory structures include an Asp-Pro-Gly (DPG) motif upstream of CRD, a less understood Thr-Thr-Xxx-Glu (TTxE) sequence, and a palmitoyltransferase conserved C-terminal (PaCCT) motif (Zaballa and van der Goot, 2018; Stix et al., 2020a; Fig. 1 A).

As transmembrane proteins, ZDHHCs localize to specific regions of the endomembrane system. Many accumulate in the Golgi (Ernst et al., 2018) and the ER (Ohno et al., 2006), but some, as ZDHHC5, are present at the plasma membrane and traffic through the endosomal system (Fig. 2). Interestingly, the localization of a given ZDHHC can change depending on the physiological state of cell, as shown for ZDHHC5, which relocalizes to distinct domains of the plasma membrane upon neuronal stimulation, through endocytic recycling (Brigidi et al., 2015; Abrami et al., 2017; Ko et al., 2019; Woodley and Collins, 2019).

As mentioned above, ZDHHCs appear to be rather potent enzymes. When studying ZDHHC6 regulation, we have indeed observed that the abundance of the most active form of the enzyme, a single S-acylated form, was tightly controlled, either through deacylation or targeting to degradation (Abrami et al., 2017). Although little is known about the regulation of ZDHHCs, three types of mechanisms are emerging. These can affect the localization, the substrate recognition, and/or the bonafide activity of the enzymes.

The first is the regulation of ZDHHC activity through posttranslational modifications (Zmuda and Chamberlain, 2020). So far, phosphorylation, ubiquitination, and S-acylation have been reported. As recently reviewed (Woodley and Collins, 2021), regulation by phosphorylation is best illustrated for ZDHHC5. ZDHHC5 is involved in numerous processes such as synaptic plasticity, cardiac function, cell adhesion, and fatty acid uptake (Wang et al., 2019). ZDHHC5 is thought to predominantly reside in the plasma membrane. It has a long C-terminal cytoplasmic domain, with multiple regulatory elements, as well as a PDZ protein–protein interaction domain. Phosphorylation on Tyr-61 near its active site can inhibit the acyltransferase activity (Hao et al., 2020), whereas src-dependent phosphorylation on Tyr-533 leads to ZDHHC5 retention at the plasma membrane in neurons via PSD95 interaction. Synaptic activity leads to the subsequent dephosphorylation of ZDHHC5, leading to its internalization and transport to dendritic shafts where it can then acylate ∂-catenin (Brigidi et al., 2015). This in turn facilitates the movement of ∂-catenin to spines where it promotes the stabilization of N-cadherin at synapses and synaptic enlargement (Brigidi et al., 2014).

ZDHHC localization and function can also be regulated by S-acylation cascades. This was first reported for ZDHHC6, which can be modified by ZDHHC16 on three cysteines within its C-terminal cytosolic SH3 domain (Abrami et al., 2017). This study showed that ZDHHC6 exists as multiple species, each carrying a specific combination of acyl chains, affecting both their enzymatic activity and their turnover rate. A second acylation cascade was reported, where ZDHHC5 is modified on its C-terminal cysteines by ZDHHC20 (Plain et al., 2020), leading to the regulation of its distribution between the plasma membrane and internal membranes (Chen et al., 2020; Ko et al., 2019; Woodley and Collins, 2019). A recent palmitoyl-proteomics study indicates that 16 out of the 23 ZDHHCs may undergo acylation, not referring to the reaction intermediate in which the fatty acid is transiently attached to the cysteine of the DHHC motif (Jensen and Larsen, 2023 *Preprint*). Four different ZDHHC regions appear to be the target of S-acylation: (a) the N-terminal cytosolic tail, (b) an acylation site in close proximity upstream to DHHC motif; (c) the PaCCT motif (Zaballa and van der Goot, 2018; Stix et al., 2020a; Malgapo and Linder, 2021; Nguyen et al., 2023); and (d) the C-terminal cytosolic domain (Abrami et al., 2017; Plain et al., 2020). Thus, future studies are bound to reveal novel regulatory S-acylation cascades.

The second mode of regulation of ZDHHC enzymes is through the interaction of accessory proteins or cofactors (Salaun et al., 2020). Pioneering studies in yeast have shown that certain ZDHHC enzymes need cofactors, with the identification that the ERF4 acyltransferase requires interaction with ERF2 (Lobo et al., 2002). The human homologs were identified as ZDHHC9, the acyltransferase of Ras, and its accessory protein GCP16, also known as GOLGA7a (Swarthout et al., 2005). GCP16 has since been found to bind other acyltransferases as well, namely DHHHC5, 9, 14, and 18 (Ko et al., 2019; Woodley and Collins, 2019; Yang et al., 2022 *Preprint*). Interestingly, some ZDHHCs exhibit “polygamic” behavior, such as ZDHHC5, which can interact with both GCP16/GOLGA7a and GOLGA7b (Ko et al., 2019; Woodley and Collins, 2019). Based on biochemical as well as cryo-EM studies, GCP16 was recently found to stabilize ZDHHC9 through interactions with the PaCCT region (Fig. 1 A; Ko et al., 2019; Nguyen et al., 2023; Yang et al., 2022 *Preprint*). Cofactors may also affect ZDHHC trafficking as shown for ZDHHC5. ZDHHC5 is retained at the plasma membrane in a Golga7b-dependent manner (Woodley and Collins, 2019), but undergoes retrograde transport when interacting with GCP16/GOLGA7a (Ko et al., 2019). We are only beginning to understand the function of accessory proteins of ZDHHC enzymes, and future investigations should shed light on the diversity of their roles.

The third mode of regulation is through transcriptional regulation. The possibility that ZDHHC enzymes can be transcriptionally regulated was revealed by our recent study on the S-acylation of the SARS-CoV-2 spike protein (Mesquita et al., 2023 *Preprint*). We found that viral infection leads to a change in the transcriptional start site of the *zdhhc20* gene, with the use of an upstream in-frame start site. This leads to the expression of an N-terminally extended enzyme, termed ZDHHC20^Long^, which localizes to the ER while its “canonical” isoform (ZDHHC20^Short^) localizes to the Golgi and displays 40 times higher acylation activity against Spike. Transcriptional regulation is unlikely to be restricted to ZDHHC20.

One still poorly understood step for most ZDHHC enzymes is how they recognize and bind their substrates. Four main factors have been proposed to provide specificity: (a) subcellular localization; (b) accessory proteins, which act as adaptors (Salaun et al., 2020); (c) recognition between transmembrane regions (Salaun et al., 2023); and (d) the presence of protein–protein interaction domains or motifs in the N-terminal and C-terminal domains of ZDHHCs. For example, ankyrin repeats are present in ZDHHC13 and 17, which mediate interaction with Huntington protein HTT (Huang et al., 2009; Lemonidis et al., 2015). This was further confirmed by the addition of the ankyrin repeats to ZDHHC3, providing it with the de novo ability to acylate HTT (Huang et al., 2009). ZDHHC17 can however also bind substrates in an ankyrin-repeat-independent fashion (Butler et al., 2023), which indicates that more substrate binding mechanisms remain to be unraveled.

#### Deacylating enzymes

The deacylation reaction is carried out by serine hydrolase superfamily members, which we will generically refer to here as acyl-protein thioesterases (APTs). Members of this superfamily adopt an α/β hydrolase fold with serine in their catalytic pocket (Long and Cravatt, 2011; Fig. 1 C). There are >200 serine hydrolases identified so far, though not all the members carry deacylation activity. The exact number of APTs is unclear. Therefore, this review will focus on APT1 and 2, ABHD17 proteins, and PPT1 (Table 1).

#### APT1 and APT2

APT1 and APT2 were initially discovered as lysophospholipases, hence their alternative names LYPLA1 and LYPLA2 (Sugimoto et al., 1996; Toyoda et al., 1999). These enzymes have clear protein-thioesterase activity in vitro and in vivo (Hedberg et al., 2011; Won et al., 2018; Zaballa and van der Goot, 2018; Zmuda and Chamberlain, 2020). APT1 and APT2 have comparable amino acid sequences (68% identical, 81% similar; Toyoda et al., 1999) and structures (RMSD = 0.878 Å; Wepy et al., 2019). It was perhaps this similarity that led to the hypothesis that both the enzymes are functionally redundant. While this may hold true for their role in lysophospholipid homeostasis (Wepy et al., 2019), the two proteins show different substrate specificities with regard to their role as de-acylating enzymes (Abrami et al., 2017, 2021; Tomatis et al., 2010). They also differ in where they accumulate in the cell, at least in tissue-cultured cells: APT1 was predominantly found inside of mitochondria (Kathayat et al., 2018), whereas APT2 accumulates on the cytosolic side of Golgi membranes (Abrami et al., 2021; Vartak et al., 2014). To note, tagging APT1 with a bulky tag such as mCitrine appears to prevent its translocation into mitochondria (Kathayat et al., 2018), leading to a similar Golgi-localization as APT2 (Vartak et al., 2014). Similar to ZDHHCs, APTs also acquire substrate specificity by virtue of sequence elements of substrates, particularly residues up- and downstream of the acylated cysteine(s) (Amara et al., 2019), and the ability of an APT to preferentially remove acyl-chains of a given length from a substrate, i.e., C16 over C18 (Won et al., 2016). This characteristic is also observed for ZDHHCs in their ability to preferentially attach different acyl chains to substrates (Greaves et al., 2017).

Intriguingly, the majority of reported APT1 and 2 substrates are found at the plasma membrane (Abrami et al., 2010; Hernandez et al., 2017; Lynch et al., 2015; Tomatis et al., 2010) or on the ER (Abrami et al., 2017; Sandoz et al., 2023). APT1 and 2 are globular soluble proteins. They were both shown to undergo S-acylation, leading to their attachment to membranes and accumulation on the Golgi for APT2 as well as APT1 (mCitrine tagged versions) when expressed in the cytoplasm. Vartak et al. (2014) proposed that APT Golgi/non-Golgi pools result from a rapid interconversion of acylated (Golgi) and non/deacylated (cytosolic) forms. This interconversion was anticipated to be mediated by the autodeacylation activity of APTs (Kong et al., 2013). Vartak et al. (2014) further hypothesized that soluble APTs are the active form of the enzymes and that the Golgi pools are required to control the overall activity and thus the steady-state amounts of acylated substrates (Vartak et al., 2014). While the proposed model is elegant, some elements are at odds with our recent findings on APT2 (Abrami et al., 2021). We found that APT2 can be acylated both by Golgi-localized ZDHHC3 and by ER-localized ZDHHC7 on its single cysteine, Cys-2. Knockdown of one of the two acyltransferases was insufficient to lose APT2 Golgi accumulation. Only a double knockdown completely abolished it. Thus, Golgi accumulation is not solely due to the acylation of APT2 on the Golgi. Furthermore, an acylation-deficient APT2 mutant, which is cytosolic, was unable to deacylate APT2 substrates. In contrast, it was shown that APT2 needs to approach the membrane, even deform it (Fig. 1 C), and stably associate with it to interact with its substrate, extract the attached acyl chain from the membrane, and ultimately hydrolyze the thioester bond (Abrami et al., 2021). The last inconsistency with the Vartak model relates to the fluorescence recovery after photobleaching (FRAP) experiments, which show that the Golgi APT2 pool is significantly more stable than the cytosolic pool (Abrami et al., 2021), inconsistent with rapid deacylation on the Golgi.

In light of these observations, we propose an alternative model for APT2 localization and function. Given that both ZDHHC3 (Golgi) and ZDHHC7 (ER) can modify APT2 at Cys-2, we suggest that APT2 Golgi accumulation occurs via at least two routes, (a) ZDHHC3-mediated APT2 acylation at the Golgi, (b) ZDHHC7-mediated APT2 acylation on the ER, followed by vesicular trafficking to the Golgi. Acylated APT2 molecules can subsequently leave the Golgi via vesicular transport to reach the plasma membrane where many of its targets reside. At the plasma membrane, APT2 will be deacylated by local thioesterase(s) that remain(s) to be identified. Such a dynamic deacylation mechanism is reminiscent of the signaling molecule Ras (Pedro et al., 2017). Once deacylated, APT2 can either recycle back to interact with ZDHHC3 or ZDHHC7 for the next cycle (this could explain the plasma membrane accumulation observed by Vartak et al. [2014]) or be degraded (Abrami et al., 2021). Future studies will challenge this working model.

#### ABHD17 proteins

Given that yeast express a single APT enzyme (Duncan and Gilman, 2002) that resembles APT1/2, these were considered the only mammalian cellular deacylation enzymes. However, the knockdown of APT1 and APT2 did not affect the acylation status of postsynaptic density protein 95 (PSD95) and N-Ras (Lin and Conibear, 2015), indicating the existence of additional thioesterases. Testing other serine hydrolases revealed that ABHD17 proteins significantly promoted the deacylation of both PSD95 and N-Ras with ABHD17A being the most efficient (Lin and Conibear, 2015). These findings were extended by the Fukata group, which found that ABHD17A, B, and C can all regulate the PSD95 acylation in neurons, but that ABHD12 and 13 also had an effect (Yokoi et al., 2016). Since then, deacylation activity was reported for ABHD4, ABHD10, ABHD12, ABDH13, and ABHD17A/B/C (Bononi et al., 2021; Cao et al., 2019; Lin and Conibear, 2015; McClafferty et al., 2020; Yokoi et al., 2016), and efforts have been undertaken to exploit their therapeutic potential (Bononi et al., 2021; Long and Cravatt, 2011; Remsberg et al., 2021).

#### PPT1 and the degradation of S-acylated proteins

The abovementioned deacylating enzymes are mostly involved in removing the acyl chains added by the ZDHHC enzymes as part of the regulatory acylation/deacylation cycle of this posttranslational modification. Degradation of acylated proteins also requires the removal of the acyl chains from the cysteines. This deacylation step generally occurs in lysosomes and is mediated by protein palmitoyl thioesterase1 (PPT1; Verkruyse and Hofmann, 1996). PPT2 also exists but was reported to be enzymatically inactive (Calero et al., 2003; Soyombo and Hofmann, 1997). Interestingly, PPT1 appears to be the most abundant of all deacetylating enzymes (Table 1). Considering it is confined to lysosomes, the volume of which is ∼50-fold smaller than that of the cytosol (Griffiths et al., 1989), its local concentration must be drastically higher.

PPT1 activity is critical for health. Homozygous or compound heterozygous mutations in the *ppt1* gene indeed lead to a devastating disease, neuronal ceroid lipofuscinose1 (NCL1; CLN1; Nita et al., 2016). CLN1 is part of the large family of lysosomal storage diseases. It is a neurogenerative disease with an early onset between 6 and 24 mo, leading to developmental regression, seizures, blindness, and death around the age of 10 (Koster and Yoshii, 2019).

PPT1 is a soluble enzyme, which is targeted to and translocated into the ER lumen via its signal sequence (Lu and Hofmann, 2006). It is then trafficked to the lumen of late endosomes and subsequently lysosomes via the mannose-6-phosphate receptor pathway. An excellent recent review summarizing the current knowledge on PPT1 activity and its possible targets as well as the consequence of PPT1 loss of function at the organism level was published by Koster and Yoshii (2019). Here, we would like to discuss an important biological aspect of PPT1 function, which we feel has been overlooked. When does PPT1 actually have access to S-acylated cysteines? Addressing this question is not only important from a mechanistic point of view but is also essential to understand the therapeutic potential of PPT1 inhibitors.

**Figure 3.**
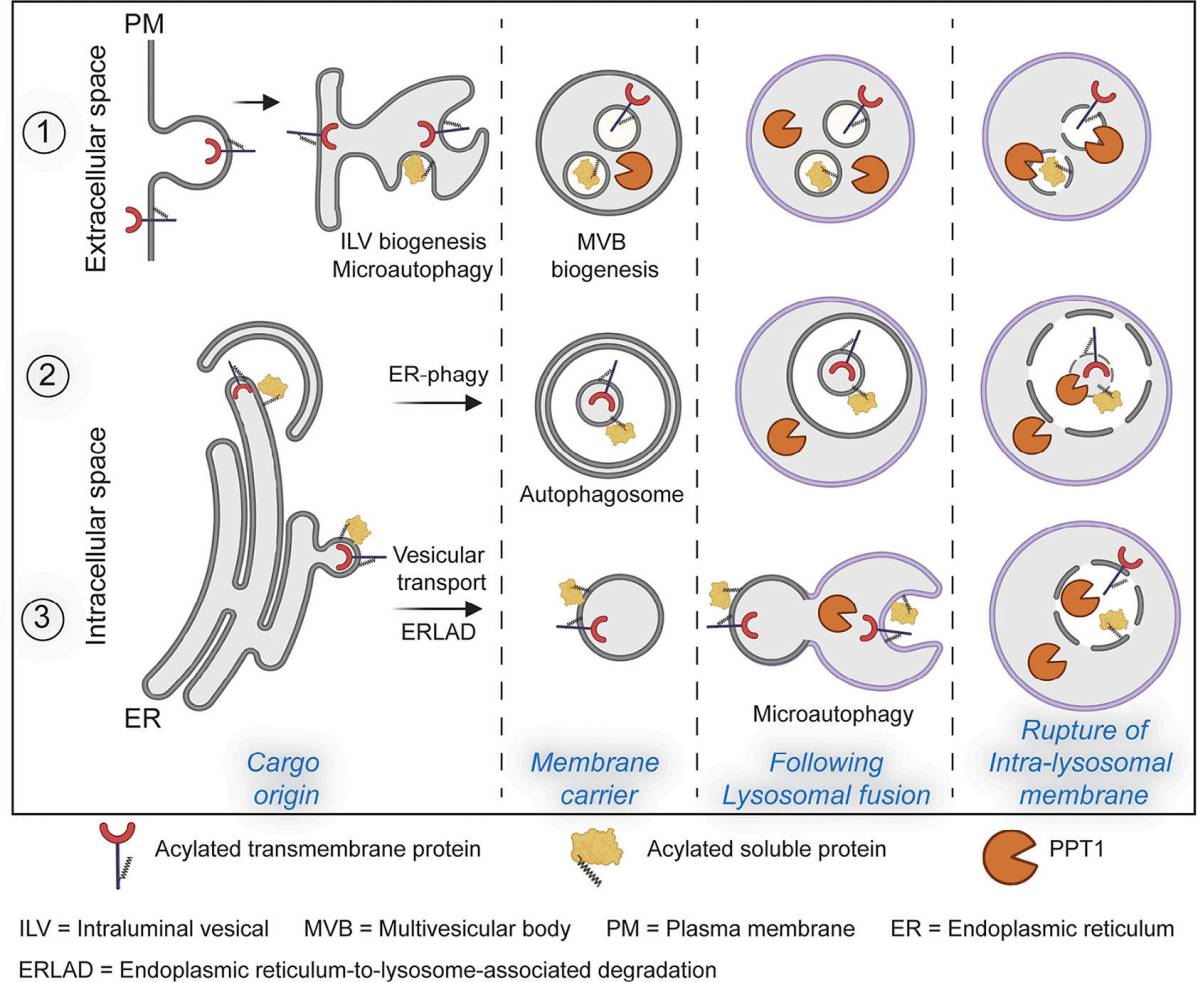
**Degradation of S-acylated proteins in lysosomes.** S-acylated proteins, whether cytosolic or transmembrane, can reach late endosomes/lysosomes via three routes: (1) by the classical endosomal trafficking route, (2) by the autophagic pathway, or (3) by interorganelle vesicular trafficking, when proteins are targeted for degradation, for example via ERLAD (ER to lysosome associate degradation; Rudinskiy and Molinari, 2023). It is only at the last step, when the membranes present in the lysosomal lumen lose their integrity, that the acylated cysteines are exposed to luminal PPT1 for deacylation. At this stage, proteins might already have been fragmented into peptides.

One must bear in mind that S-acylation occurs on cytosolic cysteines due to the cytosolic localization of the ZDHHC active sites (Fig. 1 A). To fully grasp the issue raised here, we will first describe the various trafficking routes by which S-acylated proteins can reach late endosomes/lysosomes for degradation (Fig. 3). Transmembrane (TM) proteins can reach lysosomes for degradation mainly via three routes (Fig. 3): (1) the classical endosomal trafficking route that involves the incorporation of TM proteins into nascent intraluminal vesicles (ILVs) during biogenesis of multivesicular bodies (MVBs); in this process, the cytosolic domains of proteins destined for lysosomes become incorporated in the lumen of ILVs, therefore are initially sheltered from lysosomal enzymes; (2) the autophagic pathway, where fragments of organelles to be degraded are encapsulated into a double membrane structure, whereby they are found in the lumen of the autophagosome, an already double-membraned structure. Fusion of the outermost membrane with lysosomes leads to the delivery of the organelle fragment, encapsulated in the inner-autophagosomal membrane (Yamamoto et al., 2023). Lastly, (3) the organelle-to-lysosomes vesicular trafficking pathways for degradation, where a vesicle that buds for example from the ER (Chino and Mizushima, 2020; Rudinskiy and Molinari, 2023), is transported to lysosome with which it fuses. In situations 1 and 2, the S-acylated sites of the TM proteins are thus initially not in contact with PPT1 because they are separated by one or two membranes. Only when these membranes become damaged, presumably by lysosomal lipases, can PPT1 access S-acylated cysteines. In situation 3, TM proteins are actually delivered to the lysosomal limiting membrane. Their degradation would then require access to the lysosomal lumen, perhaps via an additional microautophagy step (Kaushik et al., 2021) or the incorporation into an ILV-type structure. Very similar paths lead to lysosomal degradation of S-acylated cytosolic proteins (schematized by a yellow protein in Fig. 3). Thus, in all cases, an encounter between the S-acylated cysteines of a protein and PPT1 can only occur after the membrane rupture of the vesicular structure present in the lysosomal lumen. At that stage, the protein likely has already undergone significant luminal proteolysis by lysosomal proteases or at least is doomed to be degraded. Given this analysis, it is mechanistically obscure how inhibition or KO of PPT1 could lead to the rescue of acylated substrates, as described in the literature (Koster and Yoshii, 2019). Full understanding of the function of PPT1 and its potential as a drug target will require clarifying this topological conundrum.

Equally confusing are the findings that PPT1 might be a substrate for ZDHHC3 and 7 (Segal-Salto et al., 2016). Indeed, how a protein targeted to the ER lumen by a signal sequence can undergo acylation in the cytosol remains to be elucidated. It is possible that PPT1 has two lives, one in the lumen of the endomembrane system and the other in the cytosol. However, future studies are required to clarify these issues and grasp the full repertoire of PPT1 functions.

Of note, certain S-acylated proteins may undergo degradation via the proteasome. For example, upon synthesis of TM proteins in the ER, these might undergo S-acylation before their folding and assembly are completed. If these fail, the protein might be targeted for degradation via the ER-associated degradation (ERAD) pathway. To enter the proteasome, the protein would need to be deacylated, just as it needs to be deglycosylated. This catabolic process presumably will depend on the cytosolic deacylating enzymes such as APTs and ABHDs.

#### Sequential S-acylation of substrates

Sequential acylation of a given substrate can be viewed in at least two ways: (a) one cysteine gets acylated by a given ZDHHC enzyme, then deacylated by an APT, and the same cysteine could be the substrate for another ZDHHC, possibly at another subcellular location; (b) a given substrate could have more than one cysteine, which could be sequentially modified by the same enzyme or by different ZDHHCs, again at more than one subcellular location. Although very few studies have addressed such points, all the above situations have been observed.

The protein CLIMP-63 illustrates the first situation. It is a transmembrane protein found predominantly in the ER, where it is involved in organelle architecture, and at the plasma membrane, where it has a signaling role (Sandoz and Van Der Goot, 2015). CLIMP-63 has a single cytosolic cysteine that has been found to be a substrate for ZDHHC6 in the ER (Sandoz et al., 2023), and ZDHHC2 and 5 in the endocytic recycling pathway (Sada et al., 2019; Zhang et al., 2008). In the ER, it can undergo cycles of S-acylation–deacylation, the latter being APT2 mediated. In the non-acylated form, CLIMP-63 can travel to the plasma membrane. During transport or at the cell surface, it can be modified by ZDHHC2 or 5, leading to an increase in its surface dwell time.

ZDHHC6 is an interesting example of a protein with multiple acylation sites (Dallavilla et al., 2016). It has three acylation sites all modified by ZDHHC16, leading to the possible existence of eight different species. Initially, non-acylated ZDHHC (C^000^) undergoes acylation on any of the three acylation sites resulting in C^100^/^C010^/C^001^. The monoacylated species then go through two more cycles of acylation, first generating three intermediate C^110^/C^101^/C^011^ species subsequently leading to triple acylated C^111^ form (Abrami et al., 2017). These different ZDHHC6 species exhibit different half-lives and efficiencies (C^100^ was determined to be the most active species), suggesting that different acylation sites could incur different properties to a protein.

The existence of multiple acylation sites is extremely frequent in proteins. A recent proteomics study indicates that 18% of detected peptides contained two or more closely spaced acylated cysteines (Jensen and Larsen, 2023 *Preprint*). Beyond function, the presence of multiple sites may affect the kinetics of acylation and deacylation propensity (Dallavilla et al., 2016). For example, GAP43 can exist in a mono- or biacylated form. It was shown that monoacylated GAP43 undergoes faster deacylation as compared to a biacylated form (Tomatis et al., 2010). Similar observations were made on the ER-localized molecular chaperone, Calnexin (Lakkaraju et al., 2012; Dallavilla et al., 2016).

### Effects of S-acylation on protein localization and turnover

Some of the most frequently reported consequences of S-acylation, in addition to functional regulation (Abrami et al., 2010, 2021; Hernandez et al., 2017; Main and Fuller, 2022; Mesquita et al., 2021), are the influence on subcellular localization or targeting to specific membrane subdomains (Abrami et al., 2017, 2021; Gauthier-Kemper et al., 2014; Sandoz et al., 2023) and on protein turnover rate (Abrami et al., 2010, 2017; McCormick et al., 2008; Wang et al., 2023a).

The arguably simplest example is the targeting of a soluble cytosol protein to specific membranes or membrane domains through S-acylation and the release through deacylation. We have mentioned how this occurs for APT2, but the most classical example is Ras (Pedro et al., 2017). S-acylation also influences the trafficking and localization of transmembrane proteins in various compartments of the cell. The Wnt signaling coreceptor LRP6 requires S-acylation in the ER to exit the compartment (Abrami et al., 2008). In the absence of S-acylation, its long TM domain led to ER retention and targeting to ERAD. At the level of the Golgi, S-acylation is required for the transport of membrane proteins to the plasma membrane (Ernst et al., 2018), possibly by allowing ARF6-dependent incorporation into transport vesicles (Guo et al., 2022; Wang et al., 2023b). At the plasma membrane, S-acylation increases surface half-life, probably by inhibiting endocytic transport (Abrami et al., 2006; Sandoz et al., 2023; for review, see Jansen and Beaumelle, 2022). Finally, S-acylation can regulate the endocytic trafficking of proteins as shown for the sorting receptor, Sortilin, which is transported from endosomes to the Golgi when acylated while targeted to the lysosomes for degradation when not (McCormick et al., 2008).

Within these subcellular membranes, S-acylated proteins can be targeted to specific subregions (Abrami et al., 2003; Levental and Lyman, 2023; McBride and Machamer, 2010; Mesquita et al., 2021; Prior et al., 2003). Membranes are indeed subcompartmentalized with the presence of nanodomains enriched in specific proteins, sphingomyelin, and neutral lipids, such as cholesterol (Levental et al., 2020; Simons and Ikonen, 1997). While multiple proteins compartmentalize into lipid nanodomains in an acylation-dependent manner, the underlying mechanism remains somewhat elusive. It was proposed that perhaps it is the affinity of the acyl chain toward certain lipids or domains that drives these interactions (Blaskovic et al., 2013). Recent in vitro data support this hypothesis. Uchida and coworkers engineered a modified version of EGFP with a C-terminal palmitoyl moiety (EGFP-pal). Using cell-sized liposomes, the authors demonstrated the partitioning of EGFP-pal within the lipid-ordered phase containing saturated lipids and cholesterol. In addition, MβCD-mediated cholesterol removal prevented the internalization of the recombinant EGFP-pal (Uchida et al., 2022).

Some mysteries, however, do remain. Influenza virus hemagglutinin (HA) and vesicular stomatitis virus G-protein are widely used to label lipid-ordered and lipid-disordered domains, respectively (Havranek et al., 2021; Scheiffele et al., 1997). Surprisingly, despite their common utility as biomarkers to label two different lipid phases, both the proteins are S-acylated, suggesting that acylation can positively, negatively, or not affect, lipid-nanodomain association (Abrami et al., 2003; Veit et al., 2013). Elements that could contribute to the global understanding are the effects of the acyl-chain on the conformation of TMDs and on the local curvature of the surrounding membrane. It has recently been reported for Parkinson’s risk gene Synaptogamin (Syt11) that acylation-induced membrane curvature allows the binding of its protégé α-synuclein, which is involved in vesicle formation and trafficking (Ho et al., 2023).

As described above, most acylated proteins are eventually degraded in the lysosomes (Fig. 3). This does not concern proteins that undergo non-catabolic deacylation, which are then no longer qualified as S-acylated. Since S-acylation can affect subcellular and submembrane localization of proteins, it may have major consequences on protein turnover rate, i.e., cellular half-life. This has been observed for numerous proteins both soluble, such as APT2 (Abrami et al., 2021), and transmembrane, such as Calnexin (Dallavilla et al., 2016), CLIMP-63 (Sandoz et al., 2023), or LRP6 (Perrody et al., 2016). While the half-life is generally increased by S-acylation (Dallavilla et al., 2016; Lakkaraju et al., 2012), examples exist of S-acylation-increased degradation, such as for inflammasome component NLRP3 (Wang et al., 2023c) and monoacylated ZDHHC6 (Abrami et al., 2017).

The effect of S-acylation on trafficking and turnover rate is not only related to membrane partitioning but also to the interplay with ubiquitination (Abrami et al., 2006, 2008; Hach et al., 2013; Yount et al., 2012; Zaballa and van der Goot, 2018). S-acylation crosstalk with other modifications has been repeatedly reported (Ahearn et al., 2011; Nepal et al., 2023; Wang et al., 2023b; Yan et al., 2022), as recently reviewed (Ramzan et al., 2023; Zmuda and Chamberlain, 2020). In the context of degradation, it occurs frequently with ubiquitination, which could be mono, multi, or polyubiquitination (Dikic and Schulman, 2023). Counteracting effects of acylation and ubiquitination were, for example, reported to occur on the cell surface for the anthrax toxin receptors (Abrami et al., 2006) and in the ER for LRP6 either to promote folding (Perrody et al., 2016) or degradation (Abrami et al., 2008).

### S-acylation as a potential therapeutic target

Cells use different pathways to regulate their physiological activity and respond to the environment. These cellular pathways are hierarchical, comprising cascade(s) of proteins, and require sophisticated coordination in time and space among the different components for proper functioning. Acylation being a reversible modification provides the necessary on/off switching mechanism. It is due to this quality that the major components of these pathways, for example, LRP6–Wnt signaling (Abrami et al., 2008), TEAD4–Hippo pathway (Kim and Gumbiner, 2019; Noland et al., 2016), mTORC1–mTOR pathway (Huang et al., 2022; Sanders et al., 2019), H-Ras and N-Ras–Ras/MAPK pathway (Busquets-Hernández and Triola, 2021; Lynch et al., 2015), AKT and PCSK9–AKT pathway (Blaustein et al., 2021; Sun et al., 2022; Xiong et al., 2021), and EGFR–EGFR signaling (Burke et al., 2001; Kadry et al., 2021) are regulated by S-acylation. Such a broad involvement in these processes highlights the significance of S-acylation in health and disease. Below, we briefly explore how S-acylation can be exploited in the context of cancer and infectious diseases. S-acylation however also plays major roles in the brain, affecting memory and learning and being involved in various forms of degeneration. These topics have been covered by recent reviews and reports (Buszka et al., 2023; Ramzan et al., 2023; Wild et al., 2022).

#### Cancer

S-acylation has been of interest to the cancer field due to its importance for Ras function and the fact that mutations in Ras genes are associated with multiple cancers. Plasma membrane localization of Ras is indispensable for signal transduction downstream. Ras acylation is mediated by ZDHHC9 and has been linked with increased oncogenic activity. ZDHHC9 knockdown shows reduced cell membrane association, reduced signaling, and decreased oncogenic effect (Liu et al., 2016). Beyond Ras, in lung cancer models, S-acylation of the EGFR was found to promote its oncogenic activity (Ali et al., 2018; Bollu et al., 2015). Treatment with Orlistat, a fatty acid synthase inhibitor, diminished EGFR acylation and downstream signaling, resulting in decreased tumor growth and an increased sensitivity to anticancer drugs (Ali et al., 2018). Recently, CD36, a regulator of angiogenesis with severe implications in cancer was shown to require S-acylation for its Golgi to plasma membrane trafficking (Hao et al., 2020; Wang et al., 2023b). Single-cell transcriptomics data shows an upregulation of ZDHHC5 in pancreatic cancer. Treatment with the lipid-lowering drug Lomitapide downregulates S-acylation of anti-proliferation receptor SSTR5, thereby reducing cancer progression (Wang et al., 2023c). SCRIB signaling acts as a cancer suppressor controlling cell polarity and growth. SCRIB regulates Hippo, PI3K/AKT, and MAPK signaling (Johnson and Halder, 2014; Liu et al., 2022). S-acylation by ZDHHC7 regulates plasma membrane localization of SCRIB (Chen et al., 2016). This localization is paramount for SCRIB-mediated activation of the above-mentioned cellular pathways. Interestingly, APT2 overexpression leads to the release of Scribble into cytoplasm. In reverse, treatment with the specific APT2 inhibitor ML349 allows restoration of plasma membrane localization and downstream signaling, providing a potential drug for malignancies involving aberrant SCRIB signaling (Hernandez et al., 2017).

Finally, increased ZDHHC levels have been reported in brain tumors. In human glioma models, ZDHHC5 and ZDHHC9 overexpression are associated with lower survival rates (Chen et al., 2017; Zhang et al., 2021; Tang et al., 2022b). Surprisingly, data mining from the TCGA database shows an upregulation of APT1 in gliomas (Tang et al., 2022a). These findings go against the intuitive idea that a global hyper- or hypoacylation promotes cancer progression. Rather, these reports indicate that the fine-tuning of S-acylation/deacylation cycles is essential for health and that imbalance may lead to disease. Drugs could serve to tune this balance.

#### Infection and immunity

The COVID pandemic has increased the interest in understanding the role of acylation in viral infection. A protein interaction map of SARS-CoV-2 shows that both ZDHHC5 and GOLGA7 interact with Spike protein (Gordon et al., 2020). The knockdown of ZDHHC5 and GOLGA7 was later shown to decrease pseudovirus entry into cells (Zeng et al., 2021). In view of these findings, efforts on chemical inhibition of ZDHHC5 in cancer may extend to studying its antiviral role (Wang et al., 2023c). While ZDHHC5 may play an important role in the viral life cycle and the immune response to infection, we reported that SARS-CoV-2 Spike protein is mainly acylated by ZDHHC20 (Mesquita et al., 2021). Spike harbors 10 acylation sites within a 20-residue cytosolic stretch. The protein moreover forms trimers. This massive acylation of the glycoprotein influences the lipid composition of the virus, which has an unexpected high concentration of cholesterol and glycosphingolipids (Mesquita et al., 2021). The fact that various pathogens use lipid-nanodomains for cellular entry (Fivaz et al., 1999; Dadhich and Kapoor, 2020; Kulkarni et al., 2022) and the presence of nanodomains in viral membranes point toward S-acylation and lipid metabolism as novel therapeutic targets to fight viral infections.

Host cells are highly equipped to detect and fight invading microorganisms. The type-I interferon response provides immunity against DNA-containing pathogens. An important regulator of type-I interferon response, STING, is acylated by ZDHHC3, 7, and 15. Acylated STING localizes to lipid-nanodomains in the Golgi, where it recruits TBK1 and IRF3 to trigger the transcription of immune response genes (Lin, 2021; Mukai et al., 2016). Inhibitors blocking the acylatable cysteine residue of STING prevent downstream signaling and inflammation (Haag et al., 2018). In addition to type-I interferon response, cells recognize pathogen-associated molecular patterns (PAMPs) within the cytosol by NOD-like receptors 1 and 2 (NOD1/2). These proteins elicit NF-kB and MAPK responses downstream (Caruso et al., 2014). S-acylation of NOD1/2 by ZDHHC5 is mandatory for its membrane recruitment, bacterial sensing, and downstream signaling (Lu et al., 2019). At the cell surface, Toll-Like-Receptors (TLRs) are the detectors of PAMPs and activate antimicrobial pathways. Multiple TLRs have been reported to be acylated. Acylation-deficient mutant of TLR2 has reduced surface expression and limited the ability to respond to exogenous microbial insults (Chesarino et al., 2014). These data indicate a strong need for inhibitors and other small molecules targeting the S-acylation machinery for applications in microbial infections.

## Conclusion

The field of S-acylation has never been more exciting. Most enzymes have been identified, structures are starting to appear showing that ZDHHC and APT enzymes are potentially druggable in a specific manner, and an ever-increasing number of studies shows the importance of acylation in the control of proteins, cellular pathways, physiological processes, and as a consequence in health and disease. Yet a plethora of open questions remain. We still know little of the structure of ZDHHC enzymes other than their core conserved domains, their regulation, and how they manage to have specificity yet so many substrates (10–20% of the human proteome for just 23 enzymes). The regulatory potential makes S-acylation both a blessing and a curse. It is for this reason that cells have placed S-acylation machinery under multiple layers of regulation such as their activation/inactivation via PTMs, including S-acylation, requirement of accessory proteins for substrate recognition, and transcriptional regulation of ZDHHC enzymes under different stimuli. This generates multiple species of the same protein, different in acylation makeup and thus in biochemical properties. These cascades contain built-in quality control checkpoints, comprising APTs, to prevent ectopic S-acylation of a substrate. Perturbations in these regulatory modules result in hyper/hypoacylation of proteins leading to different physiological complications. Recent studies point out that in addition to a large-scale up-/downregulation of S-acylation, an imbalance in acylation/deacylation can also lead to complications. The years to come are bound to bring these interrogations to light.

## Supplementary Material

Table S1 shows the abundance of S-(de)acylation enzymes in human cells from multiple quantitative studies.
